# Elevating care: assessing the impact of telemonitoring on diabetes management at a cutting-edge quaternary hospital

**DOI:** 10.31744/einstein_journal/2024AO0748

**Published:** 2024-10-17

**Authors:** Tatianna Pinheiro da Costa Rozzino, Thalita Barreira Modena Cardim, Claudia Regina Laselva, Carolina de Lima Pires, Carolina Muriel Pongillo Mendonça, Milena Siciliano Nascimento

**Affiliations:** 1 Hospital Israelita Albert Einstein São Paulo SP Brazil Hospital Israelita Albert Einstein, São Paulo, SP, Brazil.

**Keywords:** Telemonitoring, Patient readmission, Adult, Diabetes mellitus, Hyperglycemia, Patient discharge, Length of stay, Hospitalization

## Abstract

**Objective:**

To assess whether post-discharge telemonitoring reduces hospital readmission in patients participating in the diabetes care program.

**Methods:**

This retrospective cohort study was conducted from June 2021 to December 2022 and included patients who were enrolled in the Diabetes Program under a hyperglycemia treatment protocol and eligible for post-discharge telemonitoring. The variables included age, sex, diagnosis, hospital stay, LACE Score, and readmission rate.

**Results:**

Among 165 patients who underwent telemonitoring, significant differences emerged in hospital readmission rates between those with and without telemonitoring (p=0.015), with a 15.4% lower readmission rate in the telemonitoring group (95%CI= 3.0-27.9%). Subgroup analyses revealed higher readmission rates in men without telemonitoring (15.2% difference; 95%CI= 0.4-30.0%; p=0.045), and in age groups ≤60 and ≥75 years without telemonitoring (24.2% difference; 95%CI= 4.5-43.9%; p=0.016 for ≤60 years; 37.1% difference; 95%CI= 9.9% to 64.2%; p=0.007 for ≥75 years). Additionally, patients with prolonged hospital stays (>7 days) without telemonitoring had higher readmission rates (19.5% difference; 95%CI= 4.5%-34.5%; p=0.011).

**Conclusion:**

This study suggests that post-discharge telemonitoring can effectively lower hospital readmission rates in diabetes management programs, potentially offering improved health outcomes, cost savings, and enhanced healthcare delivery to patients.

## In Brief

Unplanned hospital readmission is associated with negative health outcomes. Few studies have evaluated the relationship between the readmission of hyperglycemic patients and telemonitoring. Postdischarge telemonitoring may be effective in reducing readmission rates among patients enrolled in diabetes management programs.

## Highlights

■Telemonitoring reduced hospital readmissions in patients enrolled in the diabetes program by 15.4%.

■Men patients who received telemonitoring had lower readmission rates than those who did not.

■There was a difference in the readmission rate between those who underwent telemonitoring and those who did not in patients aged <60 and >75 years.

■Patients who stayed >10 days and underwent postdischarge telemonitoring had a lower readmission rate than those who did not undergo telemonitoring.

## Graphical abstract

**Figure:**
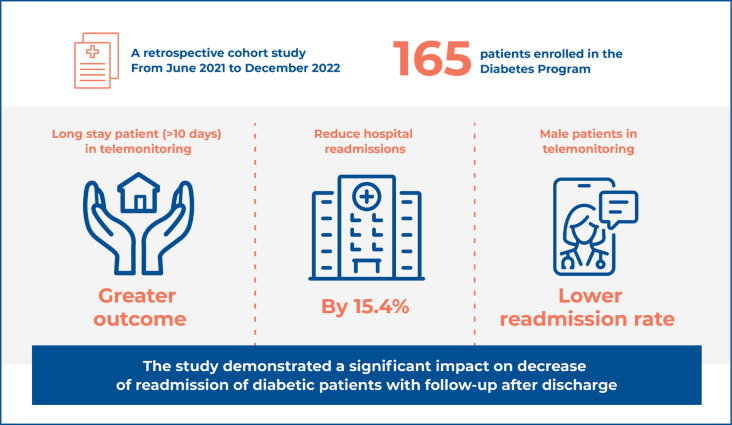

## INTRODUCTION

Unplanned hospital readmissions indicate negative health outcomes and have a significant impact on the lives of patients, families, and caregivers^(1)^ in addition to increasing healthcare system costs.^(2)^ Studies show that approximately 27% of unplanned readmissions are potentially avoidable.^(3)^ a metric often used to assess the quality of hospital care.^(4)^

Reducing the readmission rate may improve healthcare attention while simultaneously reducing costs. Furthermore, many efforts have been directed toward problem-solving to decrease this rate. Care transition strategies during the discharge process, coordination of care among healthcare providers, and interventions to improve and sustain post-discharge healthcare have been proven effective in minimizing readmissions.^(5)^

Among the various strategies used to reduce readmissions, post-discharge monitoring is a crucial care transition tool. It can effectively decrease avoidable readmissions and provide support and safety regarding self-care during the post-hospitalization phase. Some studies have demonstrated that monitoring can be conducted through phone calls, video calls, or home visits.^(6)^

Post-discharge telemonitoring has been reported to be a care transition strategy that reduces readmission rates in patients with heart failure;^(7,8)^ however, other groups of chronic diseases, such as COPD, asthma, cancer, and type 2 diabetes remained unclear.^(9,10)^

In-hospital hyperglycemia is linked to worse outcomes and is thus a marker of hospital mortality;^(11,12)^ thus, glycemic alterations are now the primary focus of diabetes care programs.

## OBJECTIVE

To assess whether post-discharge telemonitoring reduces hospital readmission in patients participating in the diabetes care program.

## METHODS

### Study type and location

This retrospective cohort study was conducted in the Chronic Patient Management (CPM) department of a private quaternary hospital in São Paulo, Brazil.

### Ethical considerations

This study was approved by the Ethics and Research Committee of *Hospital Israelita Albert Einstein* (CAAE: 61722122.8.0000.0071; #6.124.922), and the Free and Informed Consent form was granted exemption and was conducted in accordance with the recently amended 1975 Declaration of Helsinki.

### Inclusion criteria

The study included patients aged >18 years who were enrolled in a Diabetes Program and received treatment for hyperglycemia during hospitalization. The patients were also eligible for post-discharge telemonitoring. Patients who presented with glycemic changes during hospitalization (capillary blood glucose <70mg/dL or >180mg/dL) and/or requested follow-up from the doctor responsible for decompensation were eligible for monitoring using the Diabetes Program. Those who used insulin in the basal bolus regimen, according to the institutional protocol, were referred for telemonitoring.

### Data collection

All data were retrieved from an institutional operational management database containing information on patients discharged between June 2021 and December 2022.

### Protocol

Since 2018, the hospital's Chronic Patient Management Department has been utilizing post-discharge telemonitoring as a strategy. A trained multidisciplinary team uses this tool to guide care and address the potential concerns of the patients, their families, or caregivers upon returning home. The first contact occurred within 72 hours post-discharge, with subsequent contacts determined based on identified needs or being optional, as assessed by the designated healthcare professional. Up to 3 contacts should be made within the first 7-10 days post-discharge (a period with a higher rate of avoidable readmissions). The service is free of charge and incurs no additional costs to the patient.

### Clinical variables

Demographic characteristics, such as age, sex, primary diagnosis for hospitalization, length of hospital stay, LACE Score, and eligibility criteria for telemonitoring were collected from the electronic medical record.

The LACE Score is a tool developed to identify patients at high risk of hospital readmission or death within 30 days of discharge.^(13)^ It considers four variables related to hospitalization, with the first letters of each item forming the name of the score: length of stay, acute admission, Charlson Comorbidity Index, and emergency department visits in the 6 months before the current admission.

The score can range from 0 to 19, with the risk of readmission being proportional to the score. Patients with a LACE Score of 10 had a 12.2% risk of readmission or death within 30 days of hospital discharge. For every point above 10, the risk increased by at least 2%, reaching 43.7% in patients with a LACE Score of 19.

Furthermore, data related to admission date, discharge date for the length of stay calculation, and readmission within 30 days were collected.

### Statistical methods planning

Data were described using absolute and relative frequencies for categorical variables. Groups of patients who underwent telemonitoring follow-up and those who did not were compared in terms of characteristics using the χ^2^ test for categorical variables.

Associations between the need for readmission and telemonitoring follow-up, as well as potential associated factors, were investigated using generalized linear models with a binomial distribution (logistic models) using both simple and multiple approaches. The model results were presented as estimated mean proportions, 95% confidence intervals, and p-values corrected using the sequential Bonferroni method.

Analyses were conducted using the SPSS and R statistical packages with a significance level of 5%. The statistical power was estimated at 68% to detect differences between the proportions of hospital readmissions in the groups with and without telemonitoring at a significance level of 5%.

## RESULTS

A total of 165 patients who were enrolled in the hyperglycemia treatment protocol during their hospitalization and were eligible for post-discharge telemonitoring were included. Demographic characteristics were compared between patients who underwent telemedicine follow-up and those who did not. Their characteristics are presented in table 1.

**Table 1:** Characteristics and outcomes of mapped appointments for telemonitoring follow-up

Characteristics and outcomes of appointments		Total (n=165)	Telemonitoring follow-up		p value
			No (n=71)	Yes (n=94)	
Gender, n (%)					0.670#
	Female	47 (28.5)	19 (26.8)	28 (29.8)	
	Male	118 (71.5)	52 (73.2)	66 (70.2)	
Age, n (%)					0.525#
	≤60 years	71 (43.0)	27 (38.0)	44 (46.8)	
	61-74 years	57 (34.5)	27 (38.0)	30 (31.9)	
	≥75 years	37 (22.4)	17 (23.9)	20 (21.3)	
LACE Score Classification, n (%)					0.663#
	Low/moderate risk (0 to 9)	21 (17.6)	9 (19.6)	12 (16.4)	
	High risk (10 to 19)	98 (82.4)	37 (80.4)	61 (83.6)	
	Number of patients	119	46	73	
Length of stay, n (%)					0.371#
	≤7 days	43 (26.1)	21 (29.6)	22 (23.4)	
	>7 days	122 (73.9)	50 (70.4)	72 (76.6)	
Length of stay					0.740#
	≤10 days	65 (39.4)	29 (40.8)	36 (38.3)	
	>10 days	100 (60.6)	42 (59.2)	58 (61.7)	

# *χ*^2^ test.

In the assessment of the association between hospital readmission and telemonitoring follow-up, a significant difference in hospital readmission was observed; the readmission rate of patients who were not monitored through telemonitoring was higher than that of patients who underwent telemonitoring follow-up (a difference of 15.4%; 95%CI= 3.0-27.9; p=0.015). The relationship between telemonitoring and hospital readmission was explored alongside other variables of interest and is shown in table 2.

**Table 2:** Associations of hospital readmission with telemonitoring follow-up in appointments mapped for telemonitoring follow-up

Appointment characteristics		Telemonitoring	Hospital readmission estimated proportion (95%CI), %	Difference (95%CI), %	p value
All patients		No (n=71)	28.2 (19.0- 39.7)	15.4 (3.0- 27.9)	0.015#
		Yes (n=94)	12.8 (7.4-21.1)		
Gender					
	Female	No (n=19)	26.3 (11.4-49.8)	15.6 (-7.3-38.5)	0.181#
		Yes (n=28)	10.7 (3.5-28.4)		
	Male	No (n=52)	28.8 (18.2-42.5)	15.2 (0.4-30.0)	0.045#
		Yes (n=66)	13.6 (7.3-24.2)		
Age					
	≤60 years	No (n=27)	33.3 (18.3-52.7)	24.2 (4.5-43.9)	0.016#
		Yes (n=44)	9.1 (3.5-21.8)		
	61-74 years	No (n=27)	11.1 (3.6-29.3)	-8.9 (-27.5-9.7)	0.349#
		Yes (n=30)	20.0 (9.3-37.9)		
	≥75 years	No (n=17)	47.1 (25.5-69.7)	37.1 (9.9- 64.2)	0.007#
		Yes (n=20)	10.0 (2.5-32.4)		
LACE Score							
	Low/Moderate risk	No (n=9)	33.3 (11.1-66.7)	16.7 (-20.7-54.0)	0.381#
		Yes (n=12)	16.7 (4.2-47.7)	
	High risk	No (n=37)	29.7 (17.3-46.1)	15.0 (-2.2-32.2)	0.088#
		Yes (n=61)	14.8 (7.9-26.0)		
Length of stay (days)					
	≤7	No (n=21)	19.0 (7.3-41.2)	5.4 (-16.7-27.5)	0.631#
		Yes (n=22)	13.6 (4.5-34.8)		
	>7	No (n=50)	32.0 (20.6-46.0)	19.5 (4.5- 34.5)	0.011#
		Yes (n=72)	12.5 (6.6-22.3)		
	>10	No (n=42)	35.7 (22.8-51.1)	23.6 (6.9-40.4)	0.006#
		Yes (n=58)	12.1 (5.9-23.2)		

# Generalized linear models with binomial distributions (logistic models) using both simple and multiple approaches.

Values are expressed as estimated mean proportions (95% confidence intervals), and p-values were corrected by the sequential Bonferroni method.

Analysis of patients with a length of hospital stay of up to seven days and those with >7 days, a higher readmission rate in patients with hospital stays of >7 days who were not monitored through telemonitoring than in those who were monitored (a difference of 19.5%; 95%CI= 4.5-34.5; p=0.011). This difference was even greater when the length of stay was >10 days (difference, 23.6%; 95%CI= 6.9-40.4; p=0.006).

## DISCUSSION

Our study demonstrated that telemonitoring reduced hospital readmissions in patients enrolled in a diabetes program by 15% through the evaluation of post-discharge monitoring. Furthermore, men aged <60 years and >75 years, and those with a hospital stay exceeding 7 days who underwent telemonitoring also had a lower readmission rate.

Post-discharge telemonitoring is a significant care transition tool for patients with chronic diseases, including diabetes, with an impact on the quality of life and readmission reduction.^(8,14,15)^

Our study included patients directed by the Diabetes Program who were enrolled in the hyperglycemic treatment protocol during hospitalization. The literature indicates that both hyperglycemia and hypoglycemia are associated with adverse outcomes, including mortality. Thus, hospitals should focus on safe and shorter hospital stays and effective transitions to prevent acute complications and readmissions.

Hyperglycemia causes immunosuppression and vascular alterations and involves inflammatory changes that are closely related to infections. Other relevant vascular consequences of acute hyperglycemia in hospitalized patients include alterations in blood pressure, inflammation, and thrombosis.^(11)^

Patients with hyperglycemia meet more criteria for ICU admission, have longer hospital stays, and have a higher chance of unsuccessful hospital-to-home care transitions, even when compared to patients diagnosed with diabetes or normoglycemia. These factors significantly contribute to the increased morbidity, mortality, and healthcare costs associated with diabetes.^(12)^

Our results show that men who underwent telemonitoring had a 15% lower readmission rate than those who did not. This difference was not evident in the women. Sorknæs et al., studying the impact of telemonitoring in COPD patients, found that men had a hazard ratio for readmission of 2.97.^(9)^ In addition, a literature review of 34 studies found that readmission rates were higher in men than in women.^(16)^ Several studies have demonstrated differences in healthy lifestyle-seeking and preventive examinations between sexes.^(17,18)^ This difference is also reflected in the need for men to receive supervision to maintain treatment adherence, potentially elucidating the difference in readmission rates for men and the benefits of telemonitoring.^(19)^

Regarding age, our study found a difference in the readmission rate between those who underwent telemonitoring and those who did not in both age extremes studied, *i.e*., patients aged <60 and >75 years. The literature has extensively explored the higher risk of readmission in older adults.^(8,20,21)^

Despite the unexpected results presented for the younger age group, (*i.e*., <60 years) these results suggest that this age group is still economically active, and consequently, due to work commitments, has less time for self-care. In this context, our results suggest that this population would benefit from telemonitoring because telemonitoring directly influences treatment adherence.^(22)^ In addition, a study evaluating the impact of public health policies found that, in the US, the younger age group (<65 years) not targeted by public policies had significantly higher healthcare expenses for early readmissions across various pathologies.^(23)^

Regarding the LACE Score, our study demonstrated a tendency towards a significant difference between patients at a high risk of readmission who underwent telemonitoring and those who did not. However, it is convenient to assume that although our power for the total sample was close to ideal (68%), the lack of significant differences in some subgroups of patients may be due to the small number of cases with these conditions. The LACE Score has gained popularity owing to its ease of external replication and use in clinical practice, as it employs a few variables that are typically available in hospital information systems. Variable performance was demonstrated in a review. The score was developed from a cohort of patients hospitalized for clinical and surgical conditions, with an average age of 61 years. New studies have tested its utility in samples of patients with diverse epidemiological profiles and specific patient groups based on pathology. These findings suggest that the score might have a lower accuracy for certain patient groups, particularly those with a higher number of comorbidities, in contrast to the original study population composed of patients with fewer comorbidities.^(24)^

Moreover, our study demonstrated that patients who stayed for >7 days and underwent post-discharge telemonitoring had a lower readmission rate (p<0.01), in addition to presenting more relevant data from patients who stayed >10 days. Several studies have shown that longer hospital stay is associated with a higher risk of readmission.^(25,26)^ For example, Girotto et al. discussed that facilitated access to healthcare services and professionals leads to greater adherence and engagement from patients, likely because of the attention devoted to these interactions.^(27)^

It is important to highlight that despite the short intervention period presented in our study, significant readmission rates were observed for one to three contacts in the first 10 days after discharge. Short intervention duration. This brings up the discussion that even small interventions can have a major impact, and that telemonitoring may be a strategy used to reduce readmissions and consequently have better outcomes.

Our study had limitations. First, data retrieval was performed using a database from a single-center study, limiting our interpretation of patient profiles. Second, the sample size may have been relatively small, and although our power for the total sample was close to ideal (68%), no significant differences in some subgroups of patients may have been due to the small number of cases with these conditions. Third, it is possible that patients referred for telemonitoring by the Diabetes Program were more severely affected or had worse glycemic control since the program focused on decompensated patients. Unfortunately, as this was a retrospective study, the glycemic values were unknown.

## CONCLUSION

Monitoring patients after discharge through telemonitoring can be an efficient strategy for improving health outcomes, increasing patient adherence to self-care in the management of chronic diseases, and consequently decreasing readmission rates. Despite the brief and early intervention, our study demonstrated a considerable decrease in readmission of patients with diabetes at follow-up after discharge.
